# The Impaired Neurodevelopment of Human Neural Rosettes in HSV-1-Infected Early Brain Organoids

**DOI:** 10.3390/cells11223539

**Published:** 2022-11-09

**Authors:** Leonardo D’Aiuto, Jill K. Caldwell, Callen T. Wallace, Tristan R. Grams, Maribeth A. Wesesky, Joel A. Wood, Simon C. Watkins, Paul R. Kinchington, David C. Bloom, Vishwajit L. Nimgaonkar

**Affiliations:** 1 Western Psychiatric Institute and Clinic, Department of Psychiatry, School of Medicine, University of Pittsburgh, 3811 O’Hara Street, Pittsburgh, PA 15213, USA; 2 Department of Cell Biology, School of Medicine, University of Pittsburgh, 3500 Terrace Street, S362 Biomedical Science Tower (South), Pittsburgh, PA 15261, USA; 3 Academic Research Building, Department of Molecular Genetics and Microbiology, University of Florida, 1200 Newell Drive, R2-231, Gainesville, FL 32610, USA; 4 Department of Ophthalmology, University of Pittsburgh, Suite 820, Eye & Ear Building, 203 Lothrop Street, Pittsburgh, PA 15213, USA; 5 Department of Molecular Genetics and Microbiology, School of Medicine, University of Pittsburgh, 523 Bridgeside Point II, 450 Technology Drive, Pittsburgh, PA 15219, USA

**Keywords:** brain organoids, herpes simplex virus (HSV), human induced pluripotent stem cells (hiPSCs), neural rosettes, neonatal herpes encephalitis, acyclovir resistance

## Abstract

Intrauterine infections during pregnancy by herpes simplex virus (HSV) can cause significant neurodevelopmental deficits in the unborn/newborn, but clinical studies of pathogenesis are challenging, and while animal models can model some aspects of disease, in vitro studies of human neural cells provide a critical platform for more mechanistic studies. We utilized a reductionist approach to model neurodevelopmental outcomes of HSV-1 infection of neural rosettes, which represent the in vitro equivalent of differentiating neural tubes. Specifically, we employed early-stage brain organoids (ES-organoids) composed of human induced pluripotent stem cells (hiPSCs)-derived neural rosettes to investigate aspects of the potential neuropathological effects induced by the HSV-1 infections on neurodevelopment. To allow for the long-term differentiation of ES-organoids, viral infections were performed in the presence of the antiviral drug acyclovir (ACV). Despite the antiviral treatment, HSV-1 infection caused organizational changes in neural rosettes, loss of structural integrity of infected ES-organoids, and neuronal alterations. The inability of ACV to prevent neurodegeneration was associated with the generation of ACV-resistant mutants during the interaction of HSV-1 with differentiating neural precursor cells (NPCs). This study models the effects of HSV-1 infection on the neuronal differentiation of NPCs and suggests that this environment may allow for accelerated development of ACV-resistance.

## 1. Introduction

Pathogens grouped under the acronym ‘TORCH’ (*Toxoplasma gondii,* Others [syphilis, varicella-zoster, parvovirus B], Rubella virus, Cytomegalovirus, and Herpes simplex virus 1 [HSV-1] and HSV-2) [1,2] pose an important threat to the normal development of fetus or newborn.

After Rubella, HSV-1 and 2 are the next most prevalent TORCH pathogens during pregnancy [3] and represent the predominant cause of neonatal herpes and herpes encephalitis, a rare but severe disease with a mortality rate estimated to be 60% in the absence of antiviral treatment [4]. The overall rate of neonatal encephalitis is approximately 10 cases per 100,000 livebirths [4]. Although ACV therapy has drastically reduced the mortality rate in neonatal encephalitis, favorable clinical outcome in survivors is difficult to achieve even with very early diagnosis and treatment [5,6]. Long-term neurological sequela, which include varying degrees of intellectual disability, cerebral palsy, autism, cortical blindness, and epilepsy have been described in 59–69% of survivors of neonatal encephalitis [7].

HSV in neonates can be acquired during the intrauterine period (5%), peripartum period (85%), or postnatal period (10%). Peripartum and postnatal infections are categorized by their clinical manifestations that usually include: (1) SEM disease (skin, eye, and/or mouth); (2) CNS disease (central nervous system); (3) disseminated disease. The category of CNS diseases accounts for approximately one-third of neonatal encephalitis cases [8]. Intrauterine infections have the most devastating neurodevelopmental and cognitive outcomes [9]. In recent years, the investigation of the consequences of HSV infection on human brain development received a great impetus by the advances in stem cell technologies. In fact, human-induced pluripotent stem cells (hiPSC)-based technologies have revolutionized the methodological approaches to model host-pathogen interactions and offer an unprecedented opportunity to investigate multiple aspects of HSV-1 neuropathogenesis in CNS [10,11,12].

In this study, we employed early-stage organoids (ES-organoids) composed of hiPSC-derived neural rosettes-like structures. The goal of this study was to generate long-term brain organoid cultures starting from HSV-1-infected neural rosettes to investigate aspects of the potential neuropathological effects induced by the HSV-1 infections on neurodevelopment. The choice of an in vitro model based on neural rosettes relies on the fact that these structures are considered to represent the in vitro equivalent of a developing neural tube. Infected ES-organoids were cultured in the presence of ACV (50 μM) in the attempt to allow for their long-term differentiation (8–10 weeks). However, despite the antiviral treatment, infected ES organoids could not be cultured longer than 20 days. In fact, starting from day 15 post-infection (p.i.) neuronal alterations and changes in the organization of neural rosettes were observed in infected differentiating ES organoids cultured in the presence of ACV. Furthermore, infected ES-organoids exhibited loss of tissue integrity. Further analysis indicated that these changes could be attributed to the development of ACV resistance.

A second 3D model of NPCs employed in this study (adherent 3D [A-3D] [13]) showed impairment on neuronal differentiation in cultures infected at multiplicity of infection (MOI) 0.1–0.01 in the presence of ACV. Similar to what observed in ES-organoids, neuronal alterations in infected A-3D cultures could be attributable, at least in part, to the development of ACV resistance.

These results demonstrate the drastic impact that the onset of ACV resistance may have on the neuronal differentiation of NPCs. The 3D culture models of NPCs described in this study may help to (i) model neurodevelopmental defects triggered by HSV-1 infection and (ii) improve our understanding of neurological dysfunction due to neonatal HSV infection.

## 2. Materials and Methods

### 2.1. Cell Lines

Vero cells (CCL-81; ATCC; Manassas, VA, USA) for plaque assay were maintained in Dulbecco’s modified Eagle’s medium (DMEM, D6171; Millipore Sigma; St. Louis, MO, USA) supplemented with 10% fetal bovine serum (FBS, HyClone; Millipore Sigma; St. Louis, MO, USA) and 5% antibiotic/antimycotic (Anti/Anti, Gibco 15240-062; ThermoFisher Scientific; Pittsburgh, PA, USA).

Human iPSC (hiPSC) line SC0000020 (subclone SF; RUCDR Infinite Biologics; Piscataway, NJ, USA) was employed in this study. This hiPSC line was generated from fibroblasts derived from skin biopsy samples that were collected from a healthy volunteer via 4-mm full thickness punch biopsies under local anesthesia. All identifying information pertaining to the healthy volunteer was removed and the hiPSCs were established at the National Institute of Mental Health (NIMH) Center for Collaborative Studies of Mental Disorders-funded Rutgers University Cell and DNA Repository (http://www.rucdr.org/mental-health; accessed on 16 June 2021) (RUCDR Infinite Biologics, Piscataway, NJ, USA). Cells were cultured in standard conditions (37 °C, 5% CO, and 100% humidity).

SC0000020 NPCs (denoted 02SF) were generated as follows: hiPSCs were cultured in mTeSR1-plus medium supplemented with dual-SMAD inhibitors SB 431542 and LDN 193189 to promote neural induction. After 8–10 days, neural rosettes were manually isolated, transferred into Matrigel coated plates and cultured in StemDiff Neural Progenitor Medium (STEMCELL Technologies; Vancouver, BC, Canada) for the expansion of NPCs. All cells were cultured in standard conditions (37 °C, 5% CO_2_, and 100% humidity).

### 2.2. Virus Strains

The following HSV-1 strains were employed in this study: KOS (VR-1493; ATCC), and a previously detailed [14,15] KOS-based recombinant virus in which enhanced green fluorescent protein (EGFP) and monomeric red fluorescent protein (RFP) are reporters whose expression is driven by the viral promoters ICP0 and Glycoprotein C, respectively (HSV-1 DualFP).

### 2.3. Virus Preparation

The virus stocks were prepared in the D’Aiuto laboratory at the University of Pittsburgh. 80–90% confluent monolayers of Vero cells were infected at a multiplicity of infection (MOI of 3 in DMEM medium supplemented with 2% FBS. After 2 h the inoculum was removed, cells were washed and cultured for 2–3 days, until the appearance of full cytopathic effect (CPE). The cells were scraped and transferred along with the culture supernatant into 15 mL conical tubes. Cells were centrifuged at 1000 rpm for 5 min. The culture supernatant was removed, leaving behind 1.5 mL, and the cell pellet was resuspended using a vortex for 1–2 min. Cells were freeze-thawed three times. Debris was then removed by centrifuging at 3000 rpm for 5 min and the top culture supernatant containing cell-free viral particles was stored at −80 °C until use. Virus titers were determined by standard plaque assay as described below.

### 2.4. HSV-1 Plaque Assay

Plaque assays were performed after confluent monolayers of Vero cells in 24-well tissue culture dishes were achieved. Once confluent, cells were infected with serial dilutions of HSV. After infection, supernatants were aspirated, cells were washed with PBS, and one milliliter of 3% (*w*/*v*) carboxymethyl cellulose (CMC) solution overlay medium was added. Plates were incubated under standard conditions (5% CO_2_, 37 °C, and 100% humidity) for 72 h. The CMC medium was then removed, cells were washed with PBS, and finally fixed in 4% formalin solution. After one hour, the fixative was removed, and plaques were visualized by staining with gentian violet.

### 2.5. Acyclovir Resistance Assay

Rabbit skin cells (RSC) were grown on 24-well plates for plaque assays. Cells were pretreated with 50 µM acyclovir (ACV) for 4-h prior to infection. Following 4 h, cell monolayers were infected with culture supernatants for 1 h. After 1 h, the culture supernatants were removed, cells were rinsed with PBS, and media was replaced (RSC media supplemented with 50 µM ACV and IgG). Following 48 h of incubation, cells were fixed and stained with crystal violet solution (1% crystal violet, 20% EtOH). Plaques were enumerated, and data are shown as plaque forming units per milliliter (PFU/mL).

### 2.6. Generation and Infection of Early-Stage Organoids from Neural Rosettes

HiPSCs were resuspended in mTeSR1-plus medium supplemented with Rho-associated protein kinase inhibitor (ROCK inhibitor) Y27632 (STEMCELL Technologies; Vancouver, BC, Canada) and dual-SMAD inhibitors SB431542 10 µM (MilliporeSigma; St. Louis, MO, USA; S4317-5MG) and LDN193189 100 nM (Millipore Sigma; St. Louis, MO, USA; SML055-9-25MG) and seeded on Matrigel-coated plates at the density of 106 cells/well. The day after, the ROCK inhibitor was withdrawn from the neural induction medium. Culture medium was changed every day. In these culture conditions, neural rosettes make their appearance starting from day 5–7. Clusters of neural rosettes of approximately 200–300 μm were dissected manually, transferred into ultra-low attachment plates, and cultured in neural induction medium. After 2–3 days, early-stage organoids derived from cluster of neural rosettes were transferred singularly into low-attachment 96-well plates and infected as follows. Organoids were washed with 100 µL of DMEM-F12 medium. The medium was then discarded, and 10 µL of neurobasal medium with or without ACV containing 1000 pfu of HSV-1 was added. The organoids were cultured in an incubator under standard conditions (5% CO_2_, 37 °C, and 100% humidity). Two hours after the infection, the inoculum was removed, the organoids washed twice with 100 µL of DMEM-F12 medium and cultured in 5 cm petri dishes in the presence or absence of ACV.

### 2.7. Generation and Infection of Early-Stage Organoids from iPSCs

Human iPSC line SC0000020 (subclone SF) was employed to generate brain-like organoids. HiPSCs cultured with mTeSRTM plus medium (STEMCELL Technologies; Vancouver, BC, Canada) in Matrigel-coated 6-well plates were detached with Accutase and then dissociated into single cell suspension by gently pipetting. They were seeded into low attachment U-bottom 96-well plates at the density of 10,000 cells/well, in mTeSR plus medium supplemented with Rho-associated protein kinase inhibitor (ROCK inhibitor) Y27632 (STEMCELL Technologies, Vancouver, BC, Canada) to generate embryoid bodies (EBs). After three days, to induce neuroectoderm, the medium was switched to Essential 6 medium (ThermoFisher Scientific; Pittsburgh, PA, USA) supplemented with dual-SMAD inhibitors SB431542 10 µM (MilliporeSigma; St. Louis, MO, USA; S4317-5MG) and LDN193189 100 nM (Millipore Sigma; St. Louis, MO, USA; SML055-9-25MG). Cultures were observed on daily basis.

On day 8, to check whether the differentiating EBs consist of clusters of neural rosettes, randomly selected EBs were plated on Matrigel-coated 6-well plates and cultured in the abovementioned culture medium. After 24 h, visual inspection with a microscope and immunohistochemistry analysis using anti-PAX6 antibody confirmed the abundance of neural rosettes in the differentiating EBs.

On day 9, differentiating EBs were rinsed with Dulbecco’s Modified Eagle Medium: Nutrient Mixture F-12 (DMEM/F12, Gibco 11330-032) and infected individually with HSV-1 strain KOS and HSV-1 DualFP (1000 pfu/EB) in the presence or absence of acyclovir (50 μM). The average diameter (mean ± SD) of the differentiating EBs was 788.4 ± 124.5 μm. Two hours post infection, the inocula were removed, the EBs were washed with DMEM/F12, resuspended in neuronal medium (DMEM/F12 supplemented with 1× MEM Nonessential Amino Acid supplement [MEM-NEAA, CORNING^®^; Pittsburgh Corning Group, Pittsburgh, PA, USA; 25-025-CI], 1× Glutamax [Gibco; ThermoFisher Scientific, Pittsburgh, PA, USA; 35050-061], 1× N2 supplement [Gibco; ThermoFisher Scientific; Pittsburgh, PA; 17502-048], 1 µg/mL Heparin [STEMCELL Technologies; Vancouver, BC, Canada; 07980] 20 ng/mL FGF, and 20 ng/mL EGF), and supplemented or not with ACV. The culture medium was changed every other day.

### 2.8. Generation of A-3D Cultures

Monolayer cultures of NPCs were derived from hiPSC line SC0000020 (subclone SF) as previously described [14,15]. NPCs were infected as monolayer cultures at a range of low multiplicities of infection (MOI 0.1–0.0001) with HSV-1, strain KOS, in the presence or absence of ACV. Two hours after the infection, the inocula were removed, cells washed, dissociated by Accutase treatment, and seeded in optically clear 96-well plates at the density of 300,000 cells/well. Cells were cultured in StemDiff NPC medium supplemented or not with ACV (50 μM). After overnight incubation, StemDiff NPC medium was replaced with neural differentiation medium (neurobasal medium supplemented with 2% B27, BDNF 10 ng/mL, GDNF 20 ng/mL, CHIR9901 3 μM, forskolin 10 μM, dorsomorphin 1 μM, 50 U/mL penicillin G, and 50 mg/mL streptomycin) supplemented or not with ACV 50 μM. After 4 days, CHIR990, forskolin, and dorsomorphin were withdrawn. Culture medium was changed every day for the initial 7 days and afterward every other day.

### 2.9. Immunofluorescence

To prepare frozen sections, organoids were fixed in 4% paraformaldehyde (PFA) overnight at 4 °C. After three washes with PBS, organoids were immersed in 30% sucrose solution overnight. The organoids were then transferred into cryomolds (Tissue-Tek Cryo Mold Intermediate). After adsorbing traces of culture medium, the organoids were frozen in cryomolds and embedded into OCT medium at −22 °C. Sections (10 µm) were prepared by Cryostat (Micron HM350; ThermoFisher Scientific; Pittsburgh, PA, USA). Frozen sections were stored at −80 °C until needed. Before staining, frozen sections were air dried, fixed with 4% paraformaldehyde for 20 min, and incubated with 10% normal goat serum/0.2% Triton-X for 1 h at room temperature.

Samples were incubated with primary antibodies overnight at 4 °C. Primary antibodies used were mouse monoclonal anti-HSV-1 ICP4 (Abcam Cat# ab6514, dilution 1:200), rabbit polyclonal anti-SOX2 antibody (Millipore Sigma; St. Louis, MO, USA; Cat# AB5603, 1:200 dilution), rabbit monoclonal anti-nestin (Millipore Sigma; St. Louis, MO, USA: Cat# ABD69, 1:1000 dilution) mouse monoclonal anti-β-III Tubulin (R&D Systems; Minneapolis, MN, USA; Cat # NL1195V, 1:200 dilution), rabbit polyclonal anti-Microtubule-Associated Protein 2 (MAP2) (Millipore Sigma, St. Louis, MO, USA; Cat # AB5622, 1:500 dilution), and rabbit polyclonal anti-human TAU (Agilent Technologies; Santa Clara, CA, USA; Cat# A0024, 1:500 dilution). The following fluorophore-conjugated secondary antibodies were used to detect bound primary antibodies: Alexa Fluor 488 goat anti-mouse (A-10680, 1:300 dilution; ThermoFisher Scientific, Pittsburgh, PA, USA), Alexa Fluor 594 goat anti-rabbit (A-11012, 1:300 dilution; ThermoFisher Scientific, Pittsburgh, PA, USA). A fluorescent microscope (Leica CTR5500) was used for image acquisition.

### 2.10. Quantitative Confocal Analysis

Images for quantification were acquired using a Nikon A1 point scanning confocal equipped with a 10× 0.45 N.A. objective with a 4 mm working distance. Data were generated by using tool sets in both NIS Elements as well as Imaris software analysis platforms. Images were processed using Nikon’s DenoiseAI tool and segmentation of positive signal was performed using the Surface generation tool in Imaris. A nuclear count was generated by using the spot detection function in Imaris. Volumes of positive signal for TUJ1 and Nestin were reported in Imaris and were normalized to the total nuclear count in each field of view for all conditions. Representative images were generated using NIS Elements.

## 3. Results

### 3.1. Neural Rosettes-Based Culture Systems

Neural rosettes are self-organizing multicellular structures containing NPCs radially arranged a central lumen resembling an embryonal neural tube. They represent an invaluable tool to investigate normal and defective neural tube development [16,17,18].

**Figure 1:**
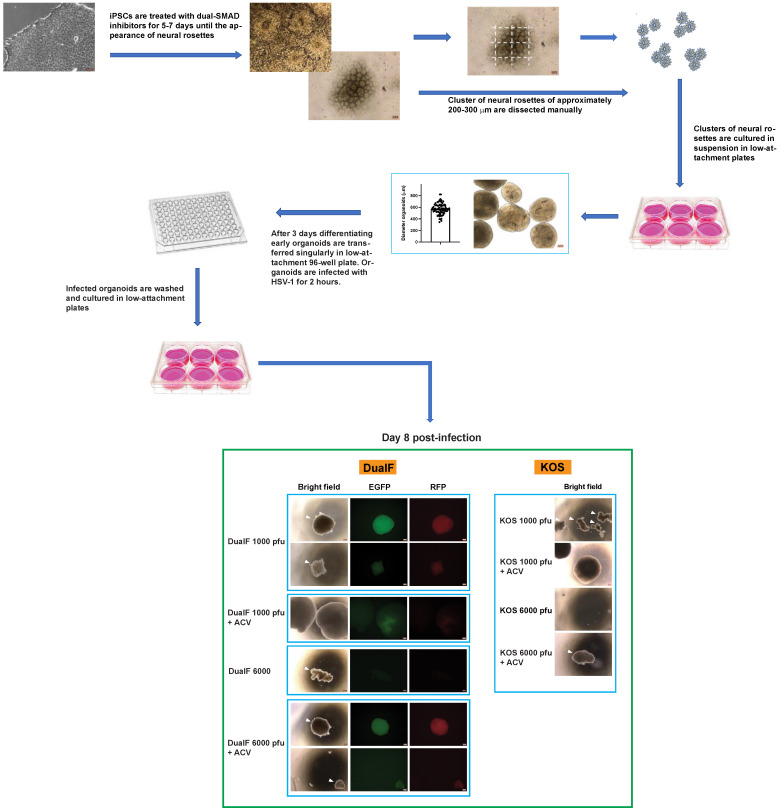
**Generation and infection of early-stage brain organoids derived from manually isolated neural rosettes.** Neural rosettes were generated by the treatment of iPSCs with dual-SMAD inhibitors. Clusters of neural rosettes were isolated manually and transferred into low attachment plates to allow for their circularization and expansion. Neural rosettes were infected individually with 1000 and 6000 pfu of HSV-1 DualFP (a KOS-based recombinant virus in which enhanced green fluorescent protein (EGFP) and monomeric red fluorescent protein (RFP) are reporters whose expression is driven by the viral promoters ICP0 and glycoprotein C, respectively) and HSV-1 strain KOS in the presence or absence of ACV. Organoids infected at higher PFU exhibited a greater degree of degradation, indicating a dose effect, and infected organoids cultured with ACV exhibited no apparent degradation at 1000 pfu but not at 6000 pfu. Together, these observations indicate a causal link between HSV-1 infection and loss of organoid structural integrity. Examples of organoids whose structural integrity was affected by HSV-1 infection are indicated by arrowheads.

We initially tested the susceptibility of neural rosettes to HSV-1 and determined a suitable viral load to model the consequence of viral infection on neuronal differentiation in the presence of ACV. Neural rosettes were derived from hiPSC line SC0000020, as described in the methods section. Briefly, hiPSCs were cultured in mTeSR1Plus medium supplemented with dual-SMAD inhibitors SB431542 (10 µM) and LDN193189 (100 nM) until the appearance of neural rosettes (5–7 days) (Figure 1). Clusters of neural rosettes of approximately 200–300 μm were dissected manually and cultured in NPS/dual-SMAD medium in ultra-low attachment plates for another 2–3 days (Figure 1).

On day 3 the mean size of the early-stage organoids derived from the cluster of rosettes (determined by measuring the diameters of 80 organoids) was 570 μm (SD = 96.37). The organoids were infected with 1000 and 6000 pfu/organoid with KOS HSV-1 strain and a KOS-based recombinant virus (HSV-1 DualF) in the presence or absence of ACV (50 μM). In DualF, the expression of EGFP and RFP are driven by the viral promoters ICP0 and Glycoprotein C, respectively [14]. The procedure to infect the organoids is detailed in the methods section. Two hours after the infection the inocula were removed, and the organoids were washed and cultured in 5 cm petri dishes in the presence or absence of ACV (Figure 1).

On day 8 p.i., all the organoids derived from rosettes infected with 1000 and 6000 pfu (KOS or DualF) had lost their structural integrity (Figure 1). Organoids infected with 6000 pfu of KOS or DualF in the presence of ACV began disaggregating to a different extent, with those infected with KOS strain showing pronounced degradation (Figure 1). Furthermore, the expression of the fluorescent reporter genes was observed in ACV-treated early organoids infected with 6000 pfu of DualF (Figure 1). Conversely, early organoids infected with 1000 pfu of KOS or DualF in the presence of ACV were intact. The presence of EGFP+ cells was limited to restricted areas in DualF-infected organoids in the presence of ACV. In the same organoids RFP+ cells were barely detected (Figure 1).

**Figure 2:**
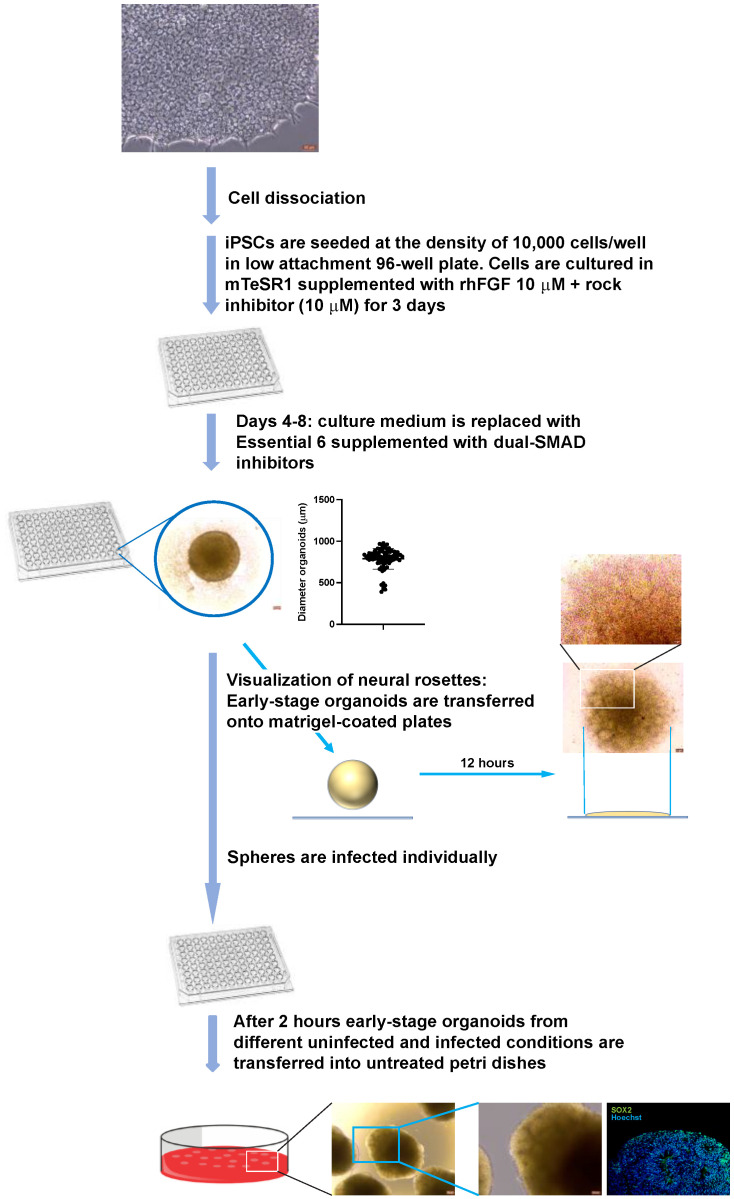
**Generation and infection of early-stage brain organoids derived from embryoid bodies. iPSCs** were seeded at the density of 10,000 cells in low attachment 96-well plate and cultured in the presence of ROCK inhibitor and rhFGF for the generation of embryoid bodies (EBs). After 3 days the EBs were exposed to dual-SMAD inhibitors for 5 days to induce the formation of neuroectoderm. After this, the differentiating EBs were mainly composed of neural rosettes. The average diameter of this early organoids (determined by measuring the diameters of 89 organoids) was 788.4 mm (SD = 124.5). Early organoids were infected individually in each well of the 96-well plates with 1000 pfu of HSV-1 strain KOS in the presence or absence of ACV. After 2 h, the inocula were removed, the early organoids washed and transferred into 10-cm untreated petri dishes for further differentiation. Microphotographs of ES-organoids after being transferred into petri dishes and staining for the NPC marked SOX2 are depicted in the bottom-most panel.

These results indicated that 1000 pfu is a reasonable amount of virus that may allow long-term differentiation of infected spheres of approximately 600 μm originating from neural rosettes in the presence of ACV, allowing the derivation of cortical organoids. However, the above outlined experimental procedure to generate and infect early-stage organoids is laborious. To simplify the experimental procedure and reduce the size variability of the spheroids containing neural rosettes, early-stage organoids were generated and infected in low attachment 96-well plate as detailed in the Materials and Methods section. Early organoids were infected individually with 1000 pfu of HSV-1 strain KOS in the presence or absence of ACV. After 2 h, the inocula were removed, the early organoids were washed and transferred into 10-cm untreated petri dishes for further differentiation (Figure 2).

**Figure 3:**
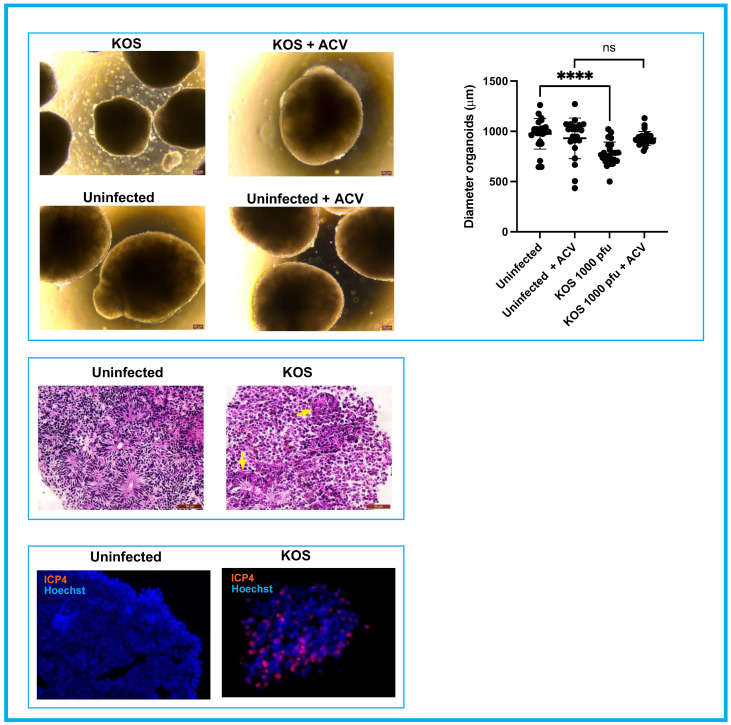
**Analysis of infected organoids on day 4 p.i**. **Top panel**: The infected cultures in the absence of ACV were significantly smaller when compared to the organoids in other conditions (Student’s *t* tests: **** *p* < 0.0001). Also, cells started detaching from the surface of these organoids indicating an initial loss of tissue integrity. Conversely, infected organoids cultured in the presence of ACV where comparable in terms of size and tissue integrity to the uninfected organoids exposed or not to ACV. **Middle panel**: Neural rosettes lost their typical organization in productively infected organoids, suggesting that the productive infection interferes with normal development of these structures. Examples of disorganized rosettes in infected organoids are indicated by yellow arrows. The expression of the viral antigen ICP4 is shown in the **bottom panel**.

On day 4 p.i., the diameter of acutely infected early organoids was significantly smaller than the uninfected ones (*p* < 0.0001). Additionally, cells detached from the surface of infected organoids and they began losing their structural integrity (Figure 3, top panel). Due to the appearance of these initial signs of structural integrity loss, productively infected organoids were fixed with PFA and processed for immunohistochemistry (IHC). IHC analysis showed the presence of cells expressing the HSV-1 immediate early gene ICP4 throughout the infected organoids (Figure 3, bottom panel), suggesting that these differentiating 3D cell structures can be efficiently infected. Hematoxylin and eosin staining showed that HSV-1 infection affected the radial organization of neural precursor cells into neural rosettes (Figure 3, middle panel).

**Figure 4:**
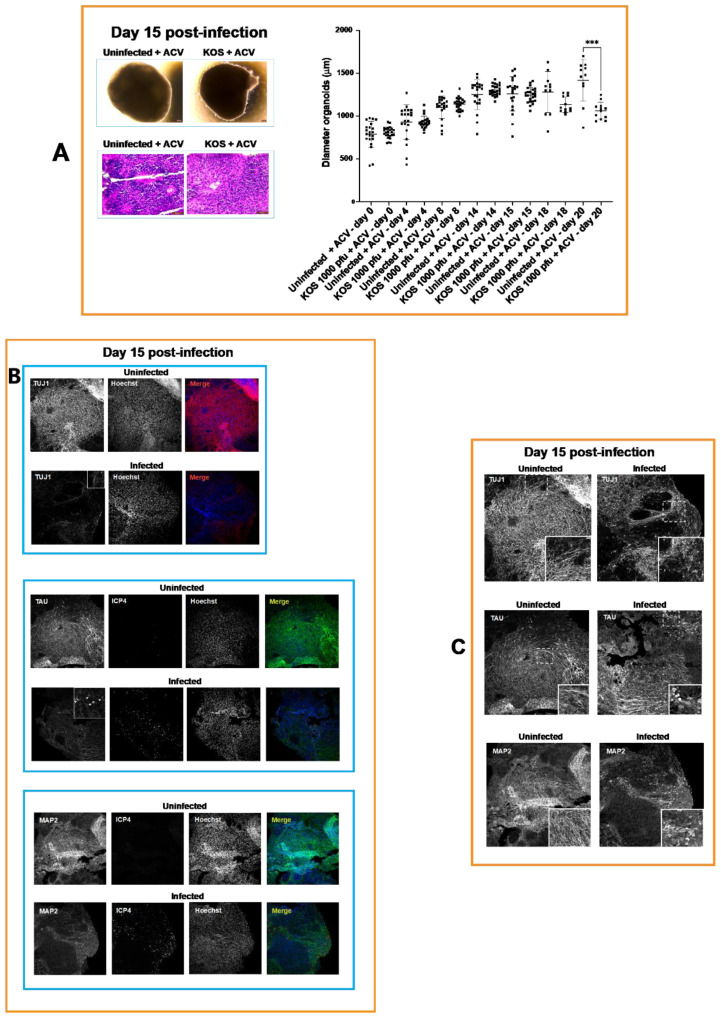
**Analysis of infected organoids on day 15 p.i.** (**A**) The infected organoids exposed to ACV began to show signs of loss of structural integrity and reduced size when compared to uninfected organoids exposed to ACV; however, this reduced size did not reach statistical significance. A significant reduction of their diameter when compared to uninfected organoids cultured in the presence of ACV (Student’s *t* tests: *** *p* = 0.0002) was observed on day 20 (see scatterplot). H&E staining highlighted the disorganization of the differentiating rosettes caused by HSV-1 infection. (**B**) The effects of HSV-1 on neuronal differentiation of infected NPCs in the presence of ACV on day 15 p.i. were analyzed by immunohistochemistry (IHC) with neuronal markers TUJ1, MAP2 and TAU. The analysis of the distribution and the expression of the TUJ1 showed the presence of neurons with very short processes. IHC of MAP2 showed reduced dendritic processes and increased perinuclear immunofluorescence. Examples of neurons exhibiting increased MAP2 perinuclear immunofluorescence are indicated by arrowheads. TAU immunofluorescence showed degeneration of neurite processes accompanied by a decreased label. Furthermore, an intense immunoreaction was observed in the soma of neuronal cells exhibiting shortening of axons (indicated by arrowheads). (**C**) In order to compare morphological features of differentiating neuronal cells in uninfected and infected organoids the fluorescence intensities were equalized. Therefore, the results in both Panels B and C indicates that ACV did not prevent neuronal alteration at day 15.

On day 15 p.i., the infected organoids exposed to ACV began to also show signs of a loss of structural integrity (Figure 2) and reduced size when compared with uninfected organoids exposed to ACV; however, this reduced size did not reach statistical significance. A significant reduction in diameter of infected organoids when compared with uninfected organoids cultured in the presence of ACV (*p* = 0.0002) was observed at day 20 p.i. (Figure 4), indicating a reduction of the ability of the differentiating cells to proliferate.

The effects of HSV-1 on neuronal differentiation of infected NPCs in the presence of ACV (50 μM) at day 15 p.i. were analyzed with IHC using neuronal markers TUJ1, MAP2 and TAU. The analysis of the distribution and the expression of TUJ1 showed the presence of neurons with very short processes. Immunohistochemistry of MAP2 showed reduced dendritic processes and increased perinuclear immunofluorescence (Figure 4). Tau immunofluorescence showed the degeneration of neurite processes accompanied by a decreased label. Furthermore, an intense immunoreaction was observed in the soma of neuronal cells exhibiting shortening of axons.

Together, these results provided evidence for the failure of ACV to prevent impairment of neuronal differentiation and loss of structural integrity in HSV-1-infected organoids.

**Figure 5:**
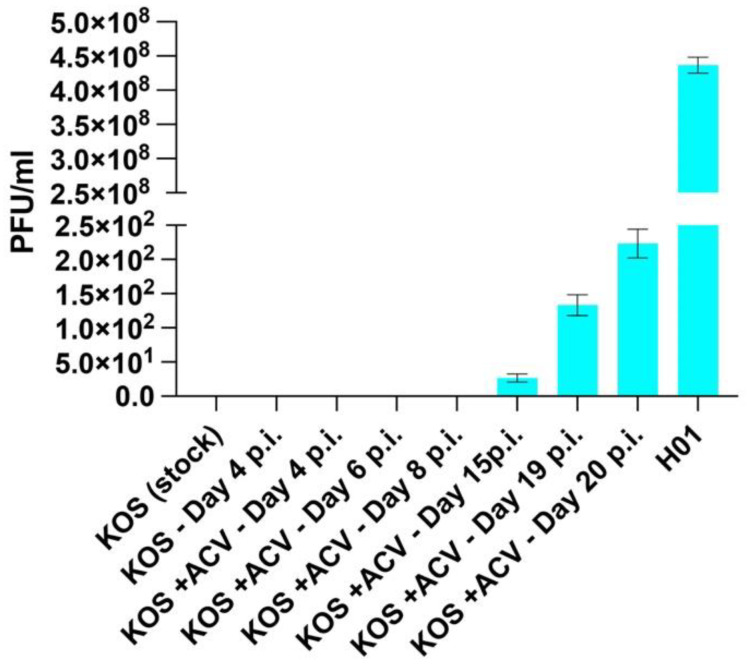
**Generation of ACV-resistance occurred during the interaction of HSV-1 with differentiating neural rosettes.** Rabbit skin cells (RSC) were grown on 24-well plates for plaque assays. Cells were pretreated with 50 µM acyclovir (ACV) for 4 h prior to infection. Following ACV pre-treatment, cell monolayers were infected with culture supernatants from days 4–20 p.i. After 1-h, the culture supernatants were removed, cells were rinsed with PBS, and media was replaced (RSC media supplemented with 50 µM ACV and IgG). Following 48-h of incubation, cells were fixed and stained with crystal violet solution (1% crystal violet, 20% EtOH). Plaques were enumerated and data is shown as plaque forming units per milliliter (PFU/mL).

Next, we investigated whether the failure of the protective effect of ACV (50 μM) at day 15 p.i. was due to the generation of ACV resistance. Standard plaque assay on rabbit skin cells indicated the presence of ACV-resistant particles in the culture media of infected organoids starting from day 15 p.i. No ACV-resistant virus was detected in the viral stock used to infect the organoids (Figure 5).

The development of ACV resistance during the interaction of HSV-1 with the differentiating organoids was also investigated on NPC monolayer cultures that were incubated with serial dilutions of culture medium collected from infected organoids at different time points (see Methods section). After 2 h, cells were washed and cultured in StemDiff NPC medium supplemented with ACV (50 μM) or R430 (a novel antiviral exhibiting efficacy toward known ACV-resistant strains) [19]. After 48 h cells were fixed and immunostained for ICP4. Immunocytochemistry analysis visualized areas containing ICP4-positive (ICP4^+^) cells in cultures exposed to supernatants collected at day 15 p.i. but not at earlier time-points. No ICP4^+^ cells were observed in cultures exposed to R430 (Supplementary Figure S1). These results further supported the plaque assay data indicating the emergence of ACV-resistance during the differentiation of infected organoids.

### 3.2. A-3D Culture System

Aside from the development of ACV resistance, it may be argued that the incomplete protective effect of ACV in infected ES-organoids may be partially attributed to an incomplete penetration of the antiviral in these 3D spheroidal structures. To test this hypothesis, we employed a recently described scaffold-free adherent 3D (A-3D) culture system of central nervous system (CNS) cells, in which we have previously shown that ACV efficiently inhibits HSV-1 infection [13]. This culture system is generated by seeding hiPSC-derived NPCs at high density in Matrigel-coated optically-clear 96-well plates. Because of their high density, NPCs self-assemble in a multilayer fashion. During the differentiation process NPCs self-organize in multilayered cell structures showing features of the ventricular/subventricular zone and neuronal cells with some features of human cortical layers. The thickness of the multilayered cell aggregates in the A-3D cultures ranges up to approximately 60 μm [13].

**Figure 6:**
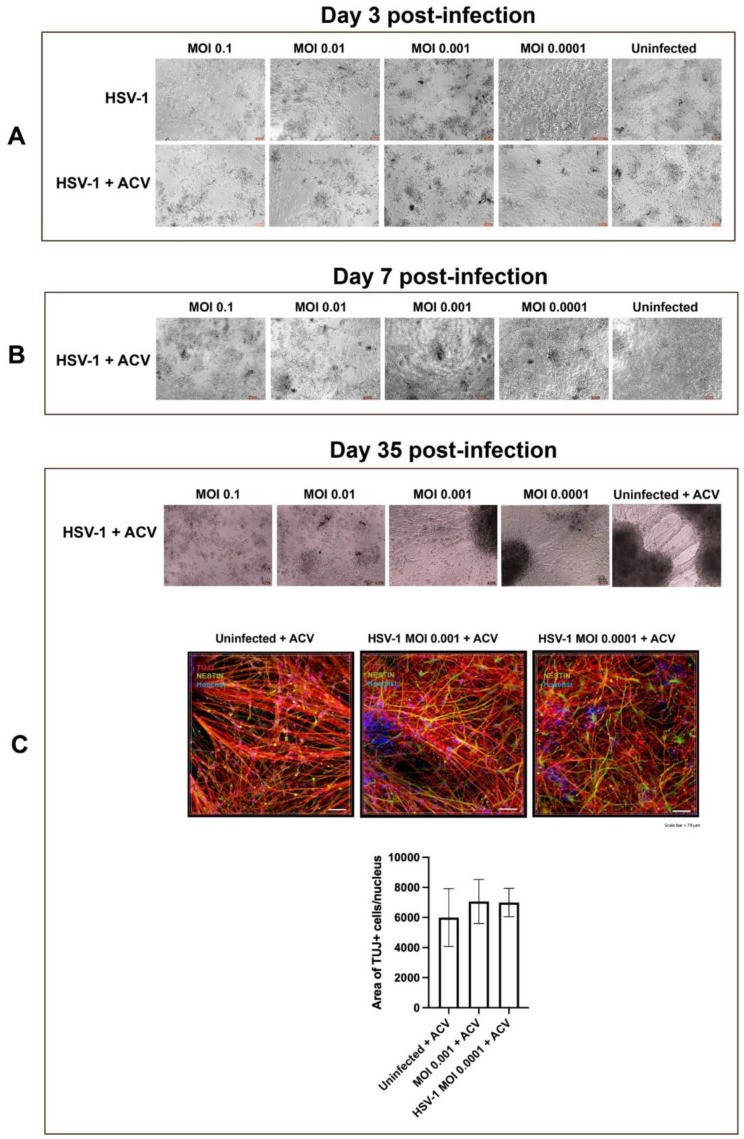
**Analysis of neuronal differentiation of NPCs in A-3D cultures in the presence of ACV.** A-3D cultures generated from monolayer cultures of NPCs were infected at a range of low multiplicities of infection (MOI 0.1–0.0001) with HSV-1, strain KOS, in the presence or absence of ACV. Microphotographs on panels A-C depict the different stages of NPCs differentiation on days 3 (**A**), 7 (**B**) and 35 (**C**). Starting from day 7, reduction of effectiveness of ACV was observed in cultures infected at MOIs 0.1–0.01 (**B**). In day 35 p.i. neuronal differentiation was observed only in cultures infected at MOIs 0.001–0.0001 cultured in the presence of ACV (**C**).

NPCs were infected as monolayer cultures at a range of low multiplicities of infection (MOIs 0.1–0.0001) with HSV-1, strain KOS, in the presence or absence of ACV. Two hours after the infection, the inocula were removed, cells washed and dissociated by Accutase treatment, and seeded in optically clear 96-well plates at the density of 300,000 cells/well. The uninfected and infected cells were cultured in StemDiff Neural Progenitor Medium supplemented or not with ACV. At day 3 p.i. in the absence of ACV, cells detached from the bottom surface of the culture wells at all MOIs, as expected from typical HSV-1 cytopathic effect (CPE) in classical permissive epithelial cell cultures (Figure 6). Conversely, in ACV-treated infected NPC cultures CPE was observed only at MOIs 0.1 and 0.01. At day 7 p.i. CPE was observed only in 2 out of 6 culture wells at MOI 0.001 but were not seen after infection at MOI 0.0001. The situation remained unchanged in the following weeks. Differentiating uninfected and infected (at MOIs 0.001–0.0001) NPC cultures were analyzed at day 35 p.i. by immunocytochemistry. Quantitative confocal analysis revealed that NPC neuronal differentiation was comparable in uninfected and infected cultures, indicating an efficacious antiviral activity of ACV at MOIs 0.001–0.0001 (Figure 6).

Next, we investigated whether the failure of the ACV antiviral activity at MOIs 0.1–0.01 could be mainly attributable to ACV-resistant mutants, similar to our observations regarding early-stage organoid cultures. To test this hypothesis, serial dilution of culture media collected from cultures at day 7 p.i. were absorbed on fresh NPC cultures. Cells were cultured in the presence or absence of ACV and R430. After 48 h, cells were fixed and immunostained for ICP4. In the presence of ACV, clusters of ICP4^+^ cells were observed only in cultures exposed to media from MOIs 0.1 and 0.01. Also, ICP4^+^ cells were detected in 2 out of 6 cultures from MOI 0.001, which showed CPE at day 7. No ICP4^+^ cells were detected in culture exposed to R430 (Supplementary Figure S2). Thus, the failure of ACV antiviral activity at MOIs 0.1–0.01 in A-3D cultures also be primarily attributable to ACV-resistant mutants.

## 4. Discussion

Neonatal HSV encephalitis is a consequence of HSV-1 fetal infection occurring during pregnancy, the birthing process or postnatally. Intrauterine HSV-1 infection, which is estimated to account approximately for 4–5% of HSV infection in neonates [9], is particularly noted for poor outcomes, including neurologic damage or death [20,21]. Despite the dramatic reduction in the mortality rate due to ACV therapy, HSV infection of the CNS remains associated with neurological complications, including poor neurodevelopmental outcomes.

The incidence of long-term neurological sequela in infants surviving neonatal encephalitis is approximately 70% [22]. Intractability of the infection to treatment caused by the generation of ACV-resistant mutants has been reported [23,24,25,26,27,28]. ACV-resistance could be generated during maternal antiviral suppressive therapy or during treatment of the neonate [29]. Although Foscarnet or vidarabine can inhibit ACV-resistant HSV infections, long-term neurological sequela remains [23].

Animal models have represented invaluable tools to investigate the development of antiviral resistance, as well as virulence and pathogenesis of the drug-resistant mutants [30]. The modeling of how neurotropic viruses affect neurodevelopment in humans has been hindered by the lack of suitable human CNS in vitro models. However, the investigation of the neurodevelopmental abnormalities caused by neurotropic viruses has received a considerable uptake with the advent of iPSC technologies (reviewed in [31]).

The goal of the present study was to model aspects of neurodevelopmental impairments which may occur during intrauterine HSV-1 infection. Specifically, we aimed to investigate, in a reductionist approach, the consequence of HSV-1 infection on the development of early-stage brain organoids (composed of clusters of neural rosettes) which mimic architectural and functional properties of early stages of the neural tube [32]. The choice of a three-dimensional (3D) instead of a two-dimensional (2D) culture model stems from evidence that 3D neural rosettes exhibit distinct epigenomic landscapes when compared to monolayer cultures of neural precursor cells [32]. Although we could have employed later stages of brain organoid development, we preferred to utilize neural rosettes (in the form of 3D spheres) because they exhibit early stages of neural development and maintain complete neural differentiation and regionalization capabilities [33]. The purpose of culturing infected ES-organoids in the presence of ACV was to protract the differentiation of these infected 3D structures for an extended period of time (9–12 weeks), which is necessary to generate brain organoids that exhibit different cortical-like structures.

The ACV treatment allowed the differentiation of early-stage brain organoids infected with 1000 pfu of HSV-1 strain KOS, up to 20 days. However, starting from day 15 p.i. the diameter of infected organoids was smaller than uninfected ones (although this difference did not reach statistical significance) and cells started detaching from the surface of these spheroidal structures (Figure 4). On day 20, the loss of tissue integrity of infected organoids was more marked, and their size was significantly smaller than uninfected organoids (Figure 4). Characterization of infected early-stage organoids exposed to ACV on day 15 p.i. showed the presence of disorganized rosettes. Further, the antiviral treatment did not prevent neuronal alteration caused by HSV-1 (Figure 4). Aspects of these neuronal alterations included retraction of neuronal processes, increased MAP2 perinuclear immunoreactivity, and strong total TAU immunoreaction in the soma of neurons with short neurite processes. These results are in line with a previous report indicating cytoskeletal disruption and retraction in HSV- infected neurons [34]. This raises the question as to whether the reduced length of the neuronal processes and the abnormal expression and distribution of the microtubule-associated proteins of MAP2 and TAU in infected organoids result from the alteration of the phosphorylation of these proteins. We hypothesize that the TAU that accumulates in the soma compartment is hyperphosphorylated, as described in Alzheimer’s disease and related tauopathies [35]. The accumulation of hyperphosphorylated TAU in the neuronal soma causes detachment of MAP2 from the microtubule [36] and alteration of MAP2 phosphorylation levels [37]. The dysfunction of MAP2 and TAU proteins affects the structure of microtubules, and it may eventually lead to the retraction of the neuronal processes and apoptosis [37,38]. Further studies are needed to investigate the dysfunction of MAP2 and TAU proteins in infected organoids.

As expected, the protective activity of ACV was viral-dose dependent. In fact, early-stage brain organoids infected with 6000 pfu and cultured in the presence of ACV exhibited loss of tissue integrity on day 8 p.i. At least two hypotheses could explain the inefficient antiviral efficacy of ACV: i) reduced ACV penetration into organoids; ii) ACV resistant HSV-1 infection. The infection of A-3D cultures ruled out the former hypothesis. In this 3D culture system, neuronal differentiation in uninfected cultures in the presence of ACV was comparable to what observed in uninfected cultures at MOIs 0.001–0.0001, but not at higher MOIs (0.1 and 0.01), where dramatic increase in CPE was obvious starting from day 7 p.i. (Figure 6). Together, the results from infected ES-organoids and A-3D cultures indicate that the risk for developing ACV-resistance in differentiating NPCs is viral dose-dependent. It should be noted that this rapid development of ACV resistance has not been described in other in vitro models of HSV infection. The fact that it is seen here likely reflects the persistent “smoldering” infection that occurs in the organoid environment that likely recapitulates the more dynamic persistence of HSV latency that exists in vivo [39].

A limitation of this study is that our 3D model did not include glial cells. We undertook a reductionist approach to focus our analyses on the interaction of HSV-1 with differentiating NPCs in neural rosettes. The evolution of our current model will consist of incorporating microglia and astrocytes into differentiating infected ES-organoids and modeling neuroinflammation at cellular and molecular levels.

In summary, our ES-organoids model shows, for the first time, neuronal alterations that may occur as a consequence of the generation and expansion of ACV-resistant mutants during neurodevelopment. The analysis of the interaction of HSV-1 with differentiating neural rosettes in a 3D environment may enable the investigation of how HSV-1 infection affects neurodevelopment and may improve our understanding of neurological sequela in survivors of neonatal herpes encephalitis.

## Data Availability

Not applicable.

## Supplementary Materials

The following supporting information can be downloaded at: https://www.mdpi.com/article/10.3390/cells11223539/s1, Figure S1: Analysis of ACV resistance developed in infected early-stage organoids using NPC monolayer cultures; Figure S2: Analysis of ACV resistance developed in infected A-3D cultures using NPC monolayer cultures.
