# Supplementary material for: The Impaired Neurodevelopment of Human Neural Rosettes in HSV-1-Infected Early Brain Organoids
Source: Cells. 2022 Nov 9;11(22):3539. doi: 10.3390/cells11223539 (PMC9688774; doi:10.3390/cells11223539)
Supplement: Supplementary file 1 [file cells-11-03539-s001.zip › cells-1984846-supplementary/Figure S2.pdf]

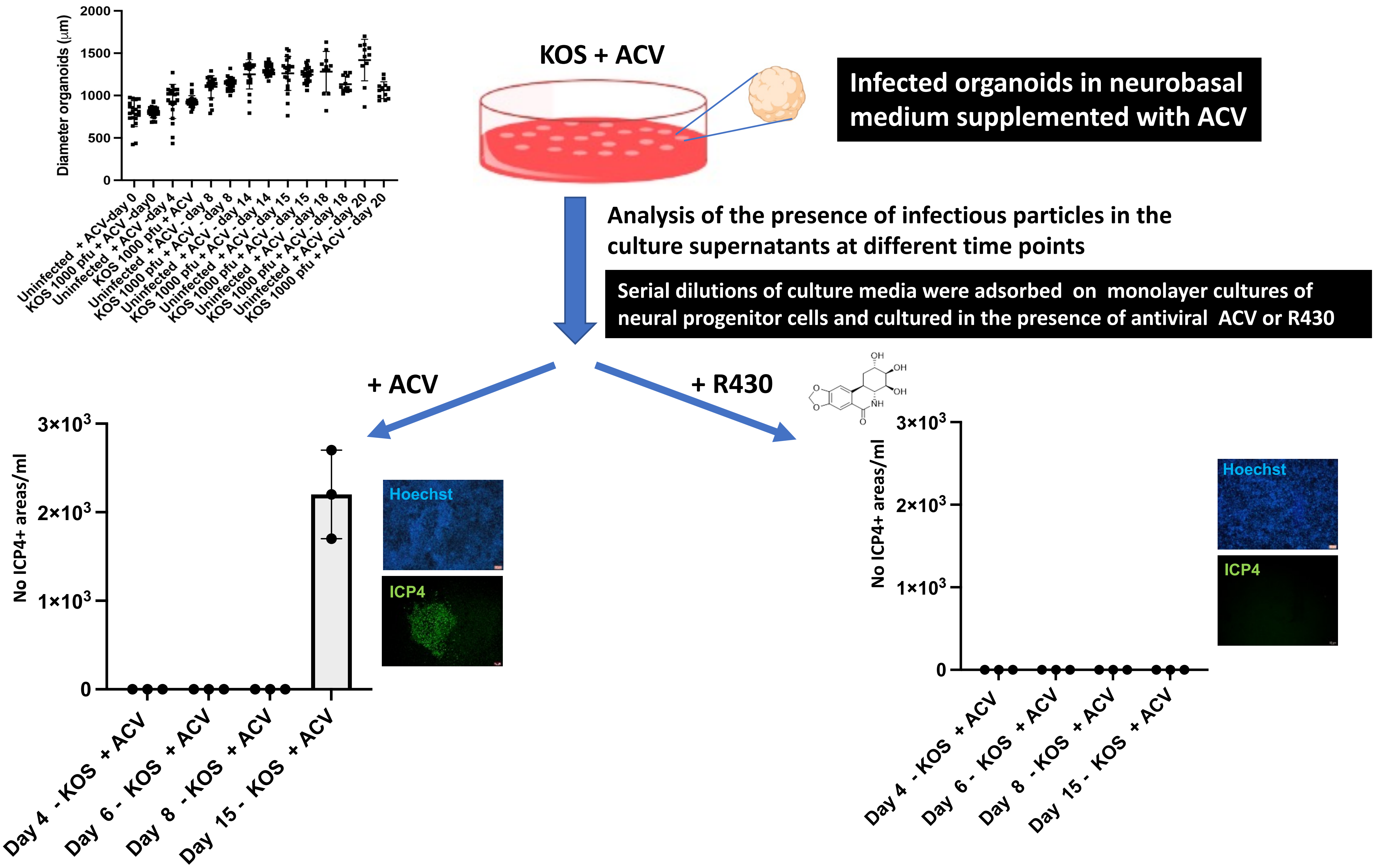

Diameter organoids (μm)
2000
1500
1000
500
0
Uninfected + ACV-day 0
KOS 1000 pfu + ACV -day0
Uninfected + ACV -day 4
KOS 1000 pfu + ACV
Uninfected + ACV - day 8
KOS 1000 pfu + ACV - day 8
Uninfected + ACV - day 14
KOS 1000 pfu + ACV - day 14
Uninfected + ACV - day 15
KOS 1000 pfu + ACV - day 15
Uninfected + ACV - day 18
KOS 1000 pfu + ACV - day 18
Uninfected + ACV - day 20
KOS 1000 pfu + ACV - day 20
KOS + ACV
Infected organoids in neurobasal medium supplemented with ACV
Analysis of the presence of infectious particles in the culture supernatants at different time points
Serial dilutions of culture media were adsorbed on monolayer cultures of neural progenitor cells and cultured in the presence of antiviral ACV or R430
+ ACV
+ R430
OH
OH
OH
NH
O
No ICP4+ areas/ml
3×10³
2×10³
1×10³
0
Day 4 - KOS + ACV
Day 6 - KOS + ACV
Day 8 - KOS + ACV
Day 15 - KOS + ACV
Hoechst
ICP4
No ICP4+ areas/ml
3×10³
2×10³
1×10³
0
Day 4 - KOS + ACV
Day 6 - KOS + ACV
Day 8 - KOS + ACV
Day 15 - KOS + ACV
Hoechst
ICP4
