# Supplementary figures and images for: The Impaired Neurodevelopment of Human Neural Rosettes in HSV-1-Infected Early Brain Organoids
Source: Cells. 2022 Nov 9;11(22):3539. doi: 10.3390/cells11223539 (PMC9688774; doi:10.3390/cells11223539)

**A**

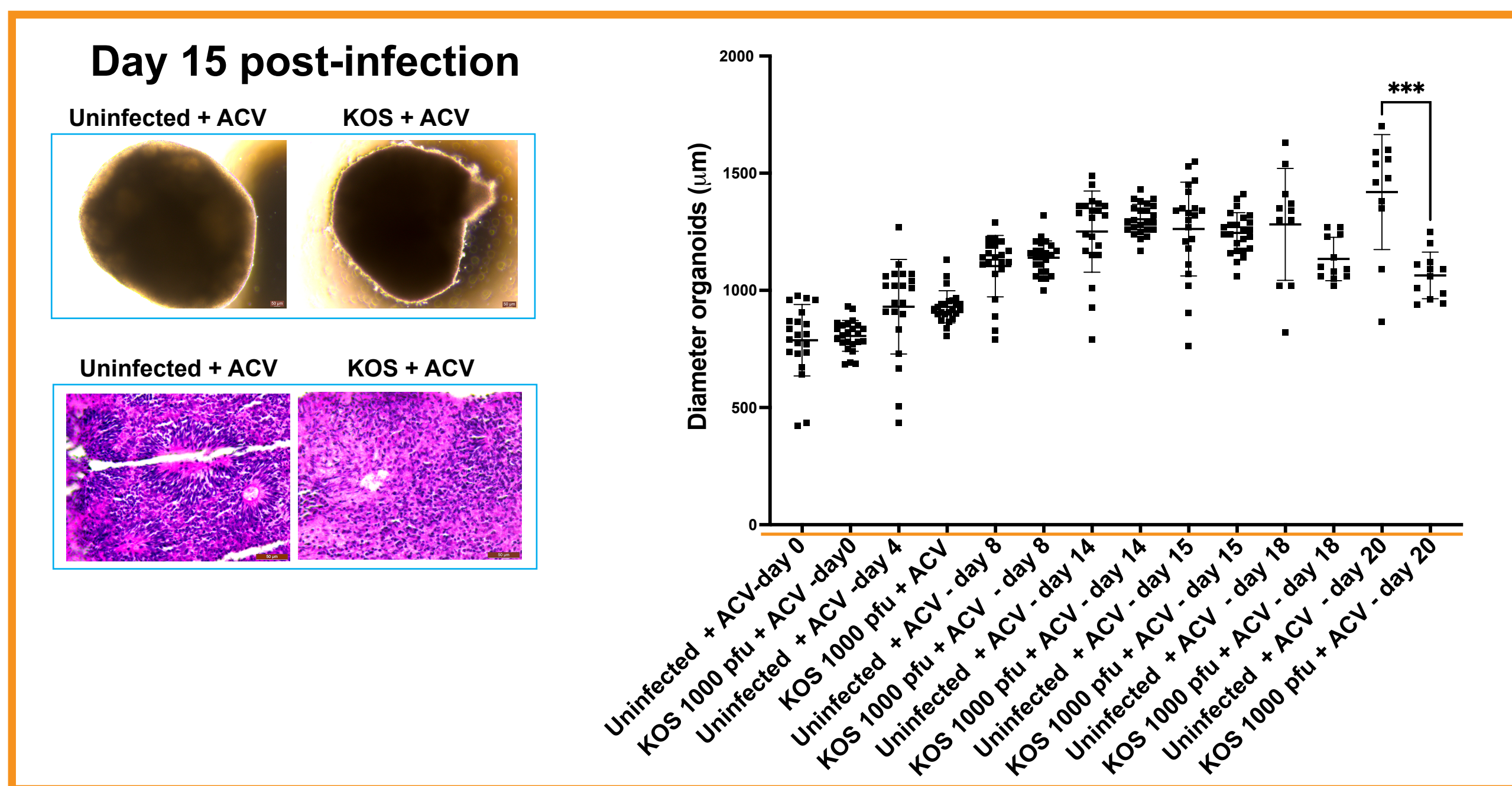

**B**

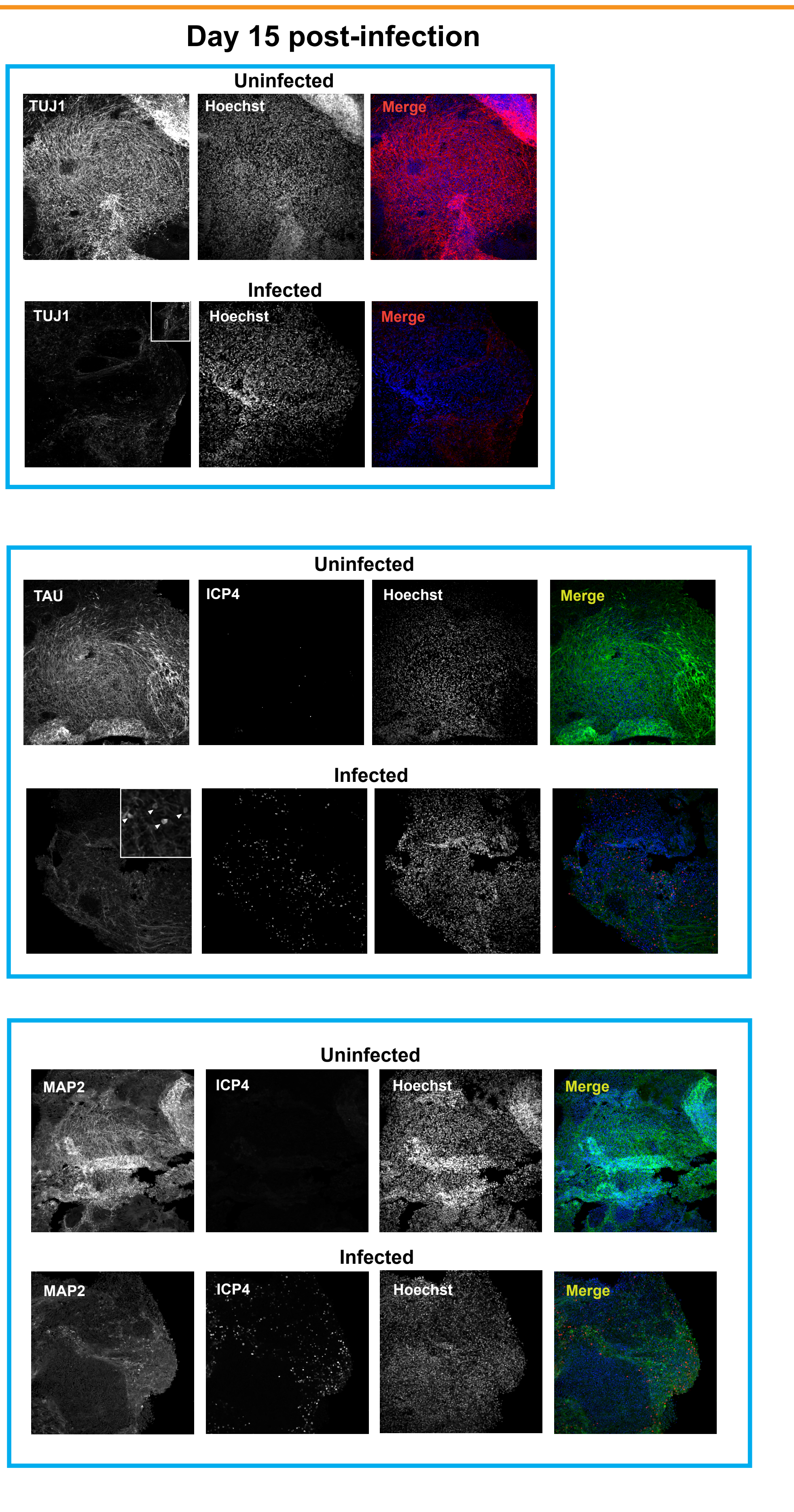

**C**

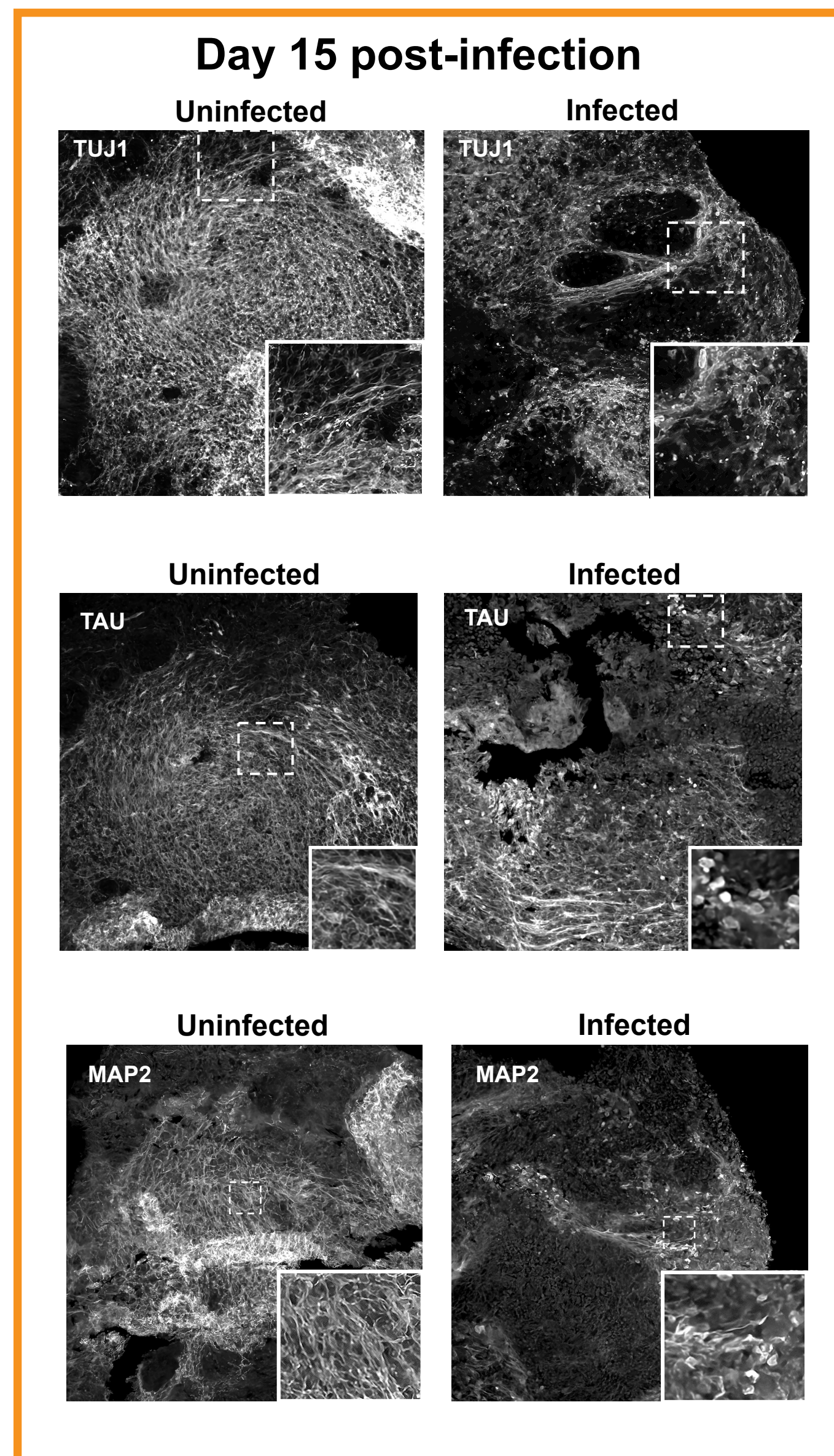

Supplement: Supplementary file 1 [file cells-11-03539-s001.zip › cells-1984846-supplementary/Figure S1.pdf]
